# Sentiment analysis of subcutaneous and intravenous immunoglobulin therapy: public healthcare perception through social media discourse

**DOI:** 10.3389/fimmu.2024.1467852

**Published:** 2024-10-10

**Authors:** Alejandro Tarango-García, Silvia Rodríguez-Narciso, Netzahualcóyotl Castañeda-Leyva, Hannia M. Prieto-Nevárez, Saul O. Lugo Reyes, Francisco J. Espinosa-Rosales, Sara Elva Espinosa-Padilla, Aidé T. Staines-Boone, Luis F. Torres-Bernal, Aristóteles Álvarez-Cardona

**Affiliations:** ^1^ Centenario Hospital Miguel Hidalgo, Aguascalientes, Mexico; ^2^ Autonomous University of Aguascalientes, Aguascalientes, Mexico; ^3^ Autonomous University of Chihuahua, Chihuahua, Mexico; ^4^ National Institute of Pediatrics (Mexico), Mexico City, Mexico; ^5^ Immunology, Allergy and Pediatrics Center, Angeles Lomas Hospital, Mexico City, Mexico; ^6^ Immunology Department, Unidad Médica de Alta Especialidad # 25, Instituto Mexicano del Seguro Social (IMSS), Monterrey, Mexico; ^7^ INOVA Vision Institute, Aguascalientes, Mexico; ^8^ Unidad de Investigación en Inmunología Clínica y Alergia Aguascalientes, Aguascalientes, Mexico

**Keywords:** natural language processing, social media, immunoglobulins, primary immunodeficiencies, sentiment analysis

## Abstract

**Purpose:**

Immunoglobulin replacement therapy remains a cornerstone of treatment in antibody deficiencies and other inborn errors of immunity. While patient preferences between subcutaneous and intravenous immunoglobulin have been studied through questionnaires, no study has yet explored patient perspectives in a free environment. Therefore, we aimed to conduct a sentiment analysis as well as a temporal and geographical analysis on public opinions obtained from social media to better understand patient satisfaction and public perception on immunoglobulin therapy.

**Methods:**

A dataset of 43,700 tweets spanning from the 1st of January of 2012 to the 31st of December of 2022 was obtained. A Valence Aware Dictionary for Sentiment Reasoning sentiment analysis was performed, followed by statistical, geographical and temporal analyses.

**Results:**

Mean polarity of intravenous immunoglobulin related tweets was 0.1295 (positive), while mean polarity for subcutaneous immunoglobulin was 0.2117 (positive). Temporal analysis through a statistical model demonstrated that the volume of tweets increased over time for both subcutaneous and intravenous treatment. Geographical analysis revealed that the majority of texts originated from the United States. The highest mean polarity was observed in Romania with a mean value of 0.2966, while the lowest polarity was documented in Norway with a mean of -0.0211.

**Conclusion:**

Tweets linked to subcutaneous immunoglobulin treatment had a higher average polarity, indicating a more positive public perception. The amount of tweets relating to both therapies showed a tendency to increase as the years progressed, implying an increase in public discussion on immunoglobulin treatment.

## Introduction

Immunoglobulin replacement therapy (IGRT) remains the cornerstone treatment for predominantly antibody deficiencies, including common variable immunodeficiency, X-linked agammaglobulinemia, and other inborn errors of immunity (1, 2). IGRT can be administered via two primary routes: subcutaneous and intravenous, each with distinct advantages and disadvantages. Subcutaneous immunoglobulin therapy is further divided into conventional and facilitated forms. The facilitated form is combined with recombinant human hyaluronidase, which increases subcutaneous tissue permeability, allowing for the infusion of larger IgG volumes and reducing the frequency of infusions compared to conventional subcutaneous immunoglobulin. The choice of administration route should be personalized, considering factors such as patient needs, clinical efficacy, number and location of infusion sites, flexibility, availability, and potential adverse reactions. For example, while intravenous immunoglobulin therapy requires fewer infusions, it offers less flexibility in terms of administration sites and is associated with a higher incidence of systemic adverse effects (3–5). Given the complexity of these considerations, it is essential not to overlook the patient’s perspective. Understanding the patient's views on IGRT can enhance the caregiver's approach to treatment, ultimately improving patient outcomes.

Social media has become a platform where patients are able to share their opinions and reviews on treatments, medications and healthcare providers. These reviews can influence the decisions of other patients, helping them to make more informed choices. Although sometimes overlooked by healthcare providers, social media has become a useful tool to learn about patients' experiences, emotional struggles and decision-making processes (6). In recent years, platforms such as X (formerly Twitter) have become an important aspect of people’s lives while also becoming an important repository for data on public opinions related to medicine (7).

In recent years, big data tools have enabled researchers to analyze the vast amounts of information generated on social media. Among these approaches, sentiment analysis has emerged as a prominent technique. Sentiment analysis involves classifying emotions expressed in text as positive, neutral, or negative by calculating a polarity score using natural language processing (NLP) models. This form of natural language processing can be classified into two main approaches, ruled-based and machine learning based (6, 8, 9). With the uprising of social media, sentiment analysis has become an optimal approach for understanding individuals' opinions on a wide range of topics, including healthcare, providing unique insights into patient care and offering a more organic way of obtaining patients' opinions on their medical experiences (10). One of those methods is VADER (Valence Aware Dictionary for Sentiment Reasoning), a lexicon and rule-based approach to sentiment analysis. VADER employs a predefined dictionary, which associates various words, lexicon features, acronyms and colloquial expressions with their corresponding positive or negative sentiments, enabling it to assess the sentiment of a given text. VADER generates a sentiment polarity score that reflects the sentiments expressed in a given text. This score ranges from -1 (indicating a more negative sentiment) to 1 (indicating a more positive sentiment), with scores closer to 0 representing neutral sentiment. VADER has been validated in previous studies using social media text and demonstrated an impressive F1 score of 0.96, outperforming individual human raters who achieved an F1 score of 0.84 (11, 12).

Currently, no studies have analyzed publicly shared experiences to understand patients’ perceptions and sentiments regarding intravenous and subcutaneous immunoglobulin therapy. This study aims to fill that gap by conducting a temporal and geographical sentiment analysis, utilizing the VADER approach, on data in the English language, retrieved from X (formerly Twitter) from the period spanning from January 1, 2012, to December 31, 2022. The present study aims to analyze social media data relating to intravenous and subcutaneous immunoglobulin therapy, with the goal of better understanding public perception on immunoglobulin replacement therapy.

## Methods

### Programming environment

The methodology was implemented using Python, a versatile programming language widely used in data analysis and machine learning. Statistical analyses were conducted using the software “R”.

### Data collection

Using Python's Tweepy library, X’s (Twitter's) API was accessed to retrieve tweets in the English language relating to immunoglobulin therapy. The search queries used were “intravenous immunoglobulin”, “intravenous immunoglobulin infusion”, “intravenous immunoglobulin replacement therapy”, “intravenous immunoglobulin therapy”, “intravenous immunoglobulin treatment”, “IVIG antibody therapy”, “IVIG”, “IVIG immunotherapy”, “IVIG infusion”, “IVIG replacement therapy”, “IVIG therapy”, “IVIG transfusion”, “IVIG treatment”, “SCIG replacement therapy”, “SCIG treatment”, “subcutaneous IgG replacement”, “subcutaneous igG therapy”, “subcutaneous igG treatment”, “subcutaneous ig infusion”, “subcutaneous ig therapy”, “subcutaneous ig treatment”, “subcutaneous immunoglobulin”, “subcutaneous immunoglobulin infusion”, “subcutaneous immunoglobulin replacement”, “subcutaneous immunoglobulin therapy”, “subcutaneous immunoglobulin treatment”, “subq ig therapy” and “subq ig treatment”. The tweets' collection was set within the date range spanning from the 1st of January of 2012 to the 31st of December of 2022. To ensure accurate and comprehensive data retrieval, only original tweets were considered, filtering out retweets. Essential fields like the creation date, geographical location, tweet text, and user information (username and location) were extracted. The gathered tweets and their associated metadata were stored in a list of dictionaries, which was then converted to a Pandas DataFrame using Python. This DataFrame was subsequently saved as a CSV file for further processing.

### Data preprocessing

Hashtags, user handles, URLs, emails, multiple spaces and special characters were removed from the tweets using functions from the neattext library. Any duplicated tweets were discarded.

### Sentiment analysis

Text sentiment analysis was performed using VADER (Valence Aware Dictionary and sEntiment Reasoner). VADER is a lexicon and rule-based sentiment analysis tool specifically designed for sentiments expressed in social media. Each tweet's sentiment was calculated using the `SentimentIntensityAnalyzer` class in VADER, and the resulting sentiment along with its polarity score were appended to the DataFrame. VADER gave a polarity score to texts that ranged from -1 (extremely negative) to +1 (extremely positive), the program then classified the tweets into three sentiment categories (13):

- Positive: For polarity score >= 0.05
- Negative: For polarity score <= -0.05
- Neutral: For scores between -0.05 and 0.05

### Temporal analysis

The tweets were further categorized based on their mention of subcutaneous immunoglobulin therapy, intravenous immunoglobulin therapy, or both. Each of these categories was further divided by year. This temporal division allows a more refined analysis of the sentiments over time in relation to the specific therapy mentioned. A statistical model was developed to explain the behavior of the number of tweets over time. This model is further explained in the “Statistical analysis” section.

### Geographical analysis

Each tweet's location was manually reviewed. Tweets containing fake locations or those whose precise location could not be reliably defined were excluded to ensure the reliability of the geographical information. Genuine locations were manually verified using the Google search engine to pinpoint the specific country of origin. For this study, only the country-level information was retained. With the sentiment polarities associated with each country, a choropleth map was created using Microsoft Excel.

### Statistical analysis

A descriptive analysis was conducted to compare the polarity between subcutaneous and intravenous replacement therapies over time. A t-test was considered for the difference in mean polarity between both therapies.

A nonlinear regression model was developed to characterize the behavior of the cumulative number of “X” social media posts per day for each type of replacement therapy. The following nonlinear model is proposed:


$$N\left(t\right)=at^{b}+\text{error fort}\geq 0$$

*N*(*t*) denotes the number of X’s messages up to time *t* ≥ 0, *a* > 0 is a parameter associated with the scale, and *b* > 0 is the power parameter. The parameters of the nonlinear model were estimated by the least squares method.

All statistical analyses were conducted using the “R” software (14).

## Results

After eliminating duplicate tweets, a total of 43,700 tweets were collected between January 1, 2012 and December 31, 2022. 43,304 tweets (99.09%) were related to intravenous immunoglobulin therapy, while 396 (.9%) were related to subcutaneous immunoglobulin therapy.

When including both therapies, there was a mean polarity of 0.1303, in which 20,585 tweets (47.1%) were positive, 11,876 tweets (27.1%) were neutral and 11,239 tweets (25.71%) were negative. From the 43,304 tweets related to intravenous immunoglobulin, there was a mean polarity of 0.1295, 20,388 tweets (47.08%) were positive, 11,707 tweets (27.03%) were neutral and 11,209 tweets (25.88%) were negative. Out of the 396 tweets related to subcutaneous immunoglobulin, the mean polarity was 0.2117, with 197 tweets (49.74%) being positive, 169 tweets (42.67%) being neutral and 30 tweets (7.57%) being negative.

**Figure 1:**
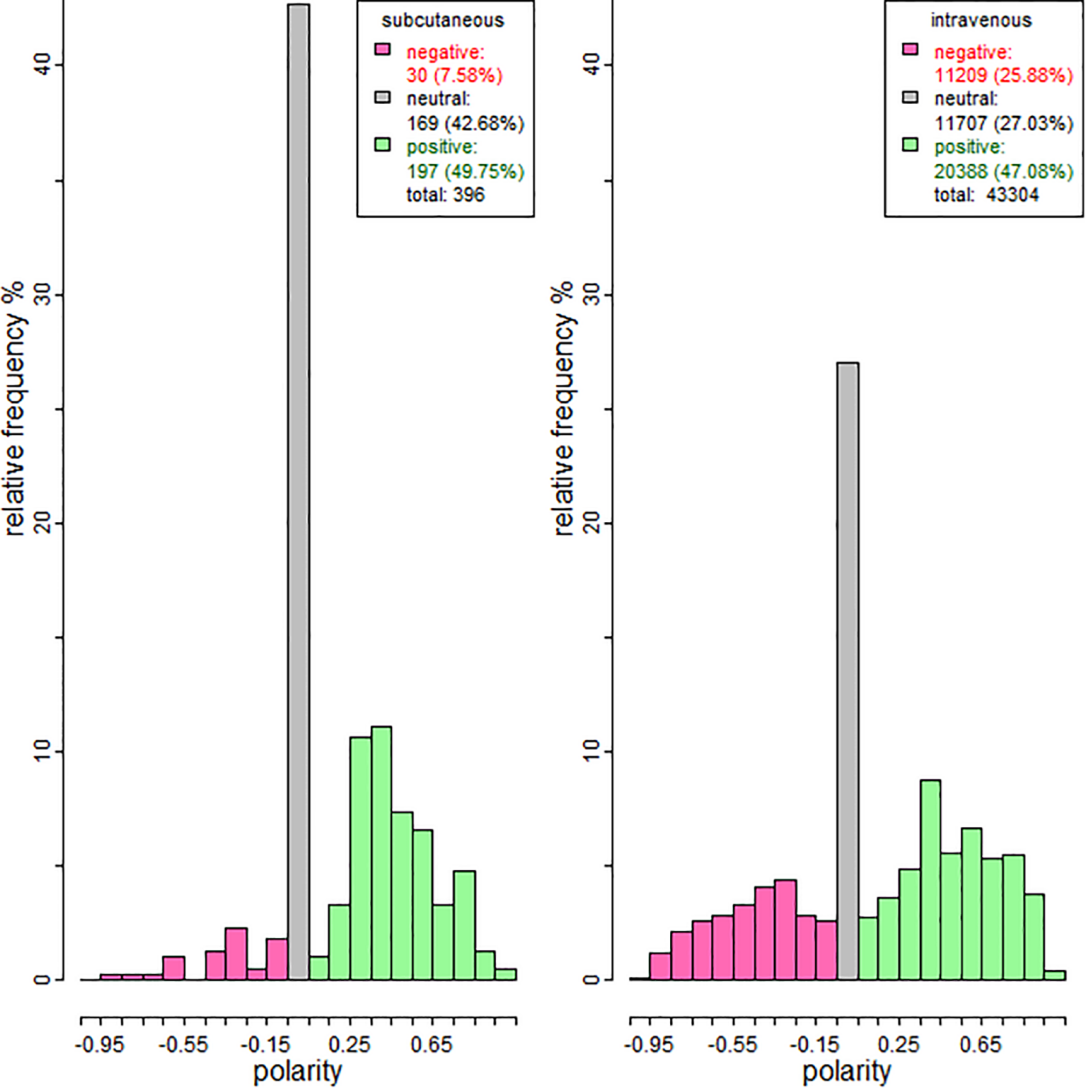
Polarity histograms of relative frequencies for subcutaneous and intravenous replacement therapies.

The neutral and positive responses to subcutaneous therapy were much more frequent relative to those related to intravenous therapy. Therefore, subcutaneous therapy is less negatively viewed upon. The shape of the distribution of both samples was skewed to the right, with multiple modes. This multimodal phenomenon is attributed to the design of the polarity classification with VADER. Both therapies show a high percentage of tweets with neutral polarity. Additionally, the number of texts with positive polarity exceeds those with negative polarity (**Figure 1**).

### Temporal analysis

**Figure 2:**
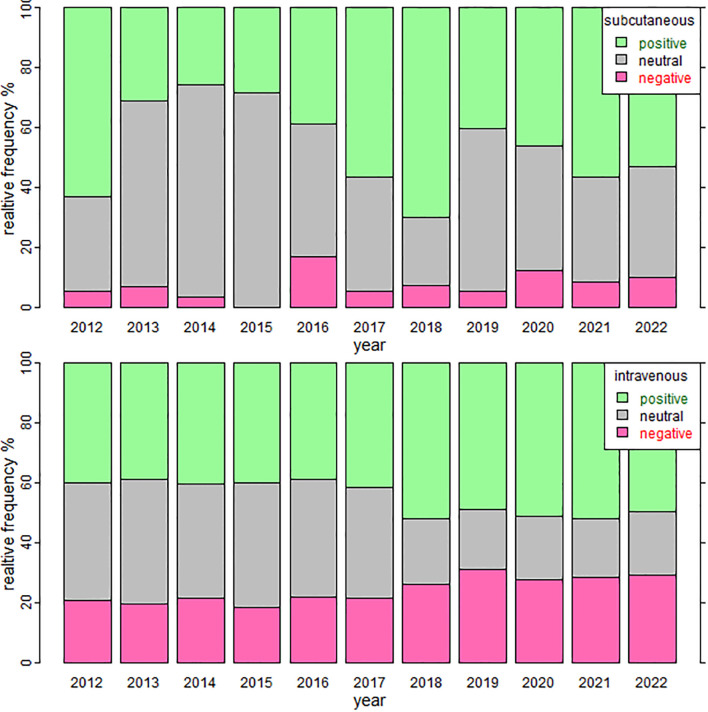
Relative frequencies of the number of negative, positive, and neutral social media texts per year for subcutaneous and intravenous therapies.

Regarding sentiment, intravenous immunoglobulin had its highest mean polarity in 2018 at 0.1597, and the lowest in 2016 at 0.0914. For subcutaneous immunoglobulin therapy, the highest mean polarity was also recorded in 2018 at 0.2996, with the lowest occurring in 2016 at 0.065. Relative frequencies of the number of negative, positive, and neutral social media texts per year for both therapies were obtained (**Figure 2**). Overall, the relative frequency of positive texts was higher than that of negative texts for both therapies. For subcutaneous therapy, there is a slight increasing trend in negative texts over time. In contrast, neutral and positive texts seem to oscillate. On the other hand, intravenous therapy showed an increase over time in both negative and positive opinions.

**Figure 3:**
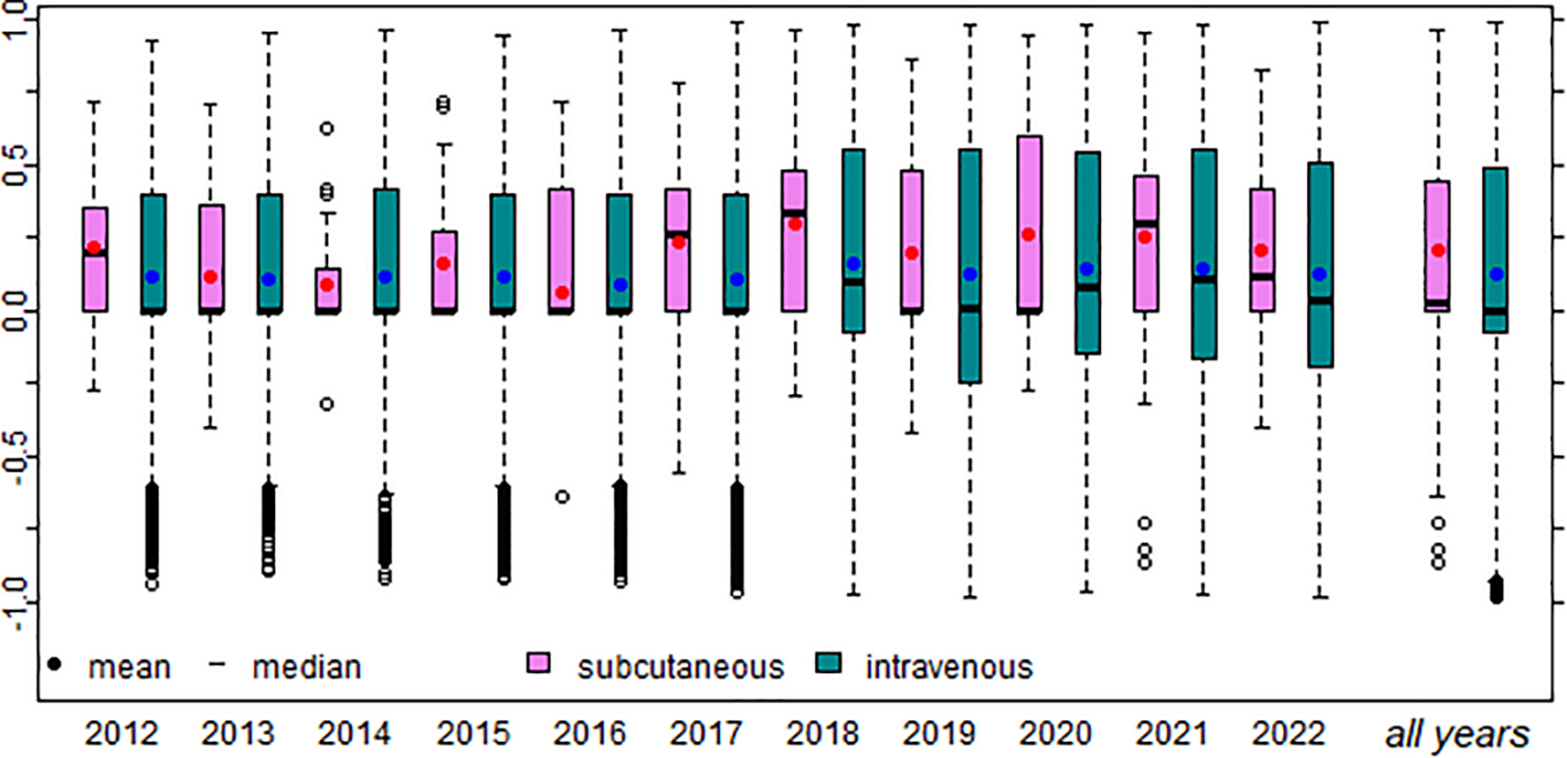
Box plot of intravenous and subcutaneous immunoglobulin replacement therapy, for each year and all years combined. The blue dots indicate intravenous mean polarity and the red dots indicate subcutaneous mean polarity.

A boxplot (**Figure 3**) demonstrated a left skew in all samples and subsamples by year. Similarly, in most years (except 2014 and 2016), subcutaneous replacement therapy is viewed more favorably than intravenous therapy. This indicates that, on average, the sentiment polarity was more positive for subcutaneous therapy.

Descriptive statistics and t-test results for the difference in means across all years between the two therapies were obtained. Given that the p-value is very close to zero (6.114e-7), it is concluded that the difference in mean polarity over all the years analyzed between subcutaneous therapy and intravenous therapy is statistically significant (**Table 1**).

**Table 1:** Descriptive statistics and Student's t-test for the difference in mean polarity over all the years between subcutaneous therapy and intravenous therapy.

replacement therapy	sample size	mean	standard error	degrees of freedom	t test	p-value
intravenous	43304	0.1296	0.0022	409.48	5.07	6.11e-07
subcutaneous	396	0.2118	0.0161			

Regarding the number of tweets on intravenous immunoglobulin, the year 2022 registered the most tweets at 7,396, and 2016 had the fewest at 2,171. Meanwhile, in relation to subcutaneous immunoglobulin therapy, 2021 witnessed the highest tweet volume at 60, while 2015 had the lowest at 14 (**Table 2**).

**Table 2:** Number of tweets per year related to intravenous immunoglobulin and subcutaneous immunoglobulin.

year	tweets related to subcutaneous immunoglobulin	tweets related to intravenous immunoglobulin
2012	19	2400
2013	29	2492
2014	31	2393
2015	14	2311
2016	18	2171
2017	39	2922
2018	57	3395
2019	37	4579
2020	41	6713
2021	60	6532
2022	51	7396
Total	396	43304

**Figure 4:**
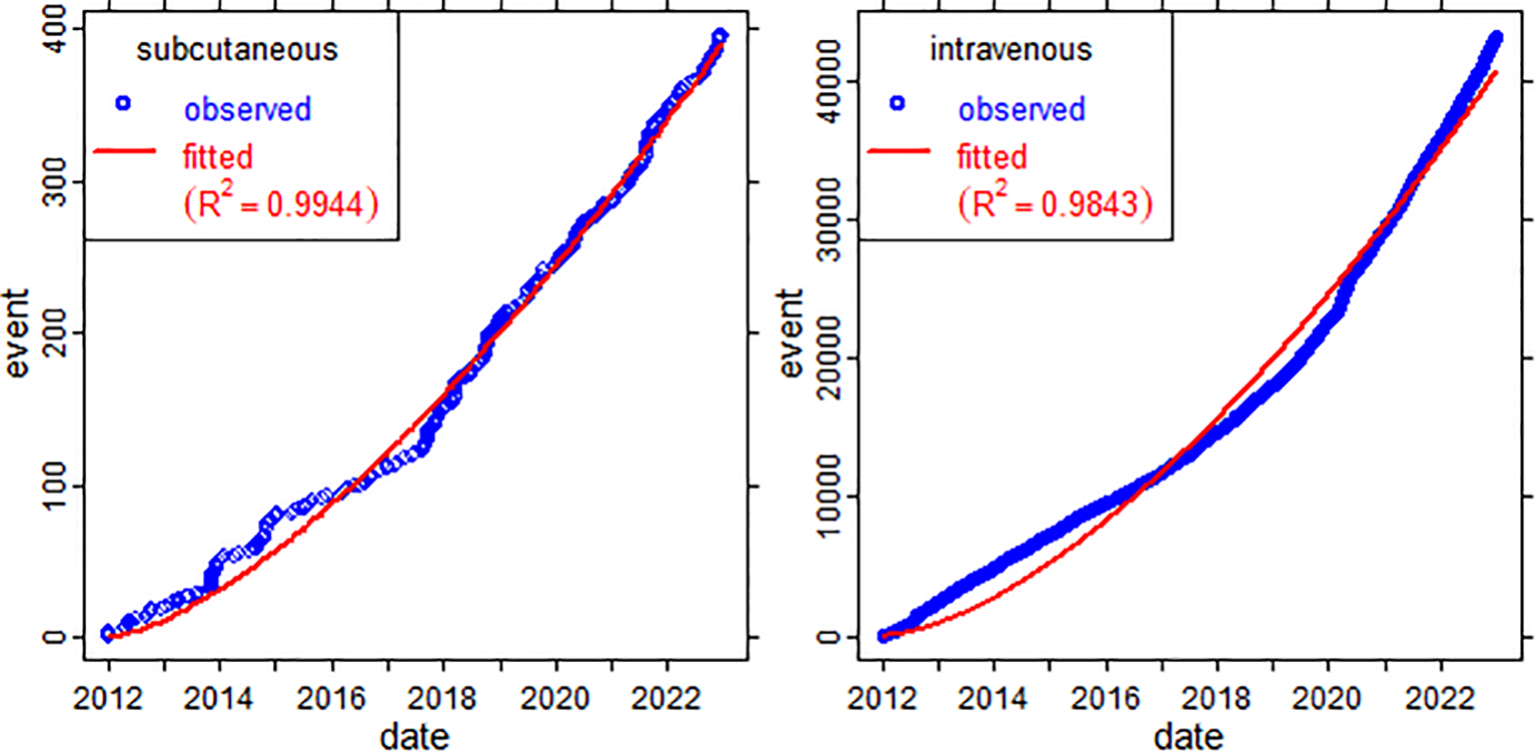
Temporal plot of the number of X’s tweets related to subcutaneous and intravenous therapies, with adjustment of the non linear regression model.

The nonlinear regression model shows a strictly increasing trend in both intravenous and subcutaneous therapy (**Figure 4**). Parameters of the nonlinear model were estimated using the method of least squares (**Table 3**). Due to the magnitude of the parameter estimates from the model, there will be much greater discussion in the long term about intravenous therapy compared to subcutaneous therapy. In both cases, the coefficient of determination is close to one.

**Table 3:** Estimated parameters of the non linear regression model.

therapy \ estimated parameter	*a*	*b*	coeffcient of determination *R^2^*
subcutaneous	0.0018	1.4782	0.9944
intravenous	0.0866	1.5738	0.9843

### Geographical analysis

**Figure 5:**
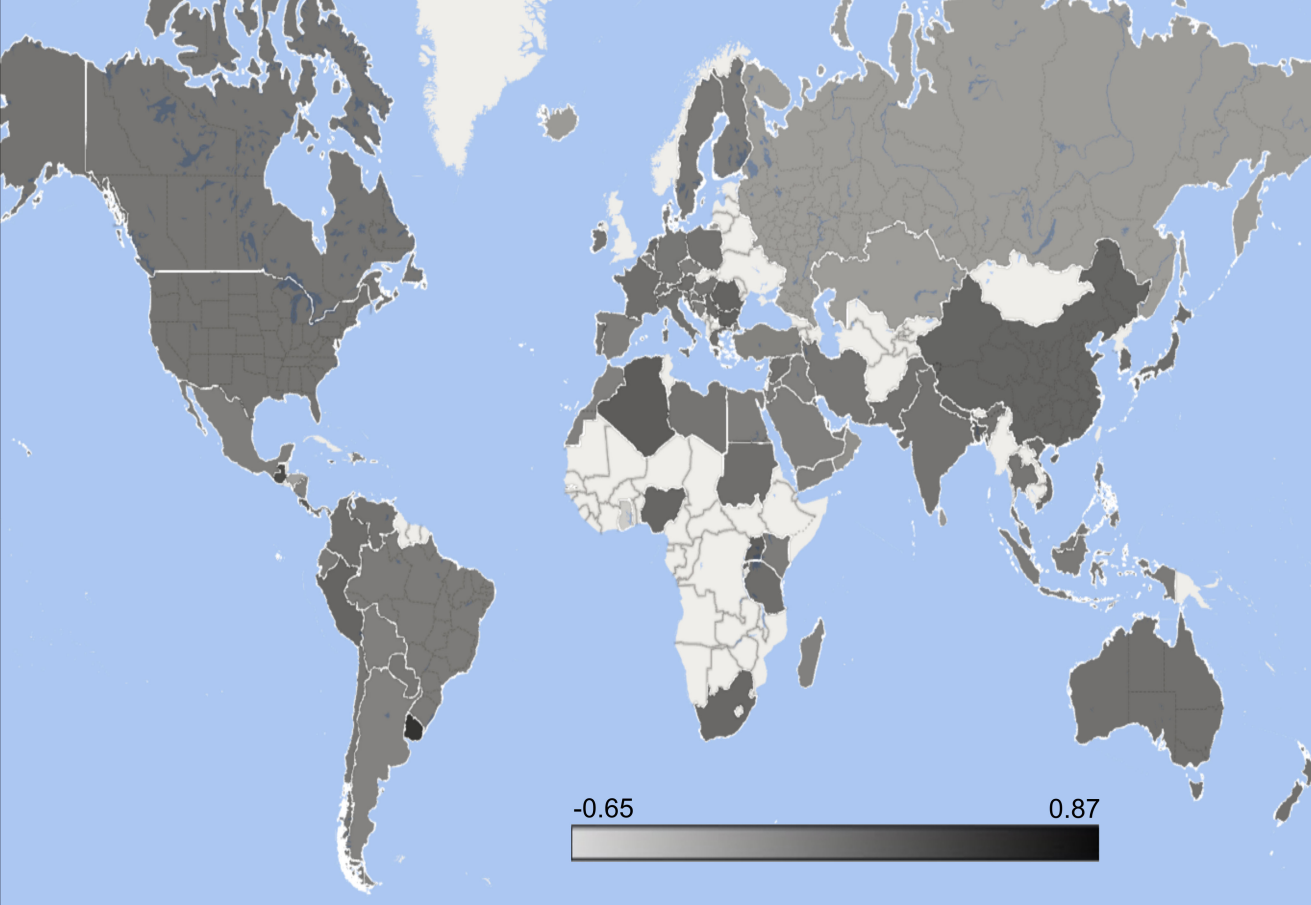
Heat map of countries by polarity on subcutaneous and intravenous immunoglobulin combined.

A total of 102 countries were identified in the analysis. The United States of America exhibited the highest tweet volume at 15,594 and 22 countries, including Luxembourg, Uruguay, Maldives, Guatemala, The Bahamas, Saint Kitts and Nevis, Costa Rica, Bangladesh, Rwanda, Lebanon, Bolivia, Bosnia and Herzegovina, Dominican Republic, Gambia, Iraq, Jordan, Madagascar, Morocco, Nicaragua, Puerto Rico, Yemen, and Kazakhstan, each recorded the least amount of tweets, with only one tweet per country (**Table 4**). The highest mean polarity, considering countries with a minimum of 10 tweets, was observed in Romania with a mean value of 0.2966, while the lowest polarity was documented in Norway with a mean of -0.0211 (**Figure 5**).

**Table 4:** Average polarity and number of tweets of the 10 countries with the highest amount of tweets.

Country	Number of tweets	Mean polarity
U.S.A.	15594	0.1402
Canada	1553	0.1172
England	1167	0.1155
India	711	0.1362
Australia	619	0.1682
Israel	301	0.1311
Spain	257	0.0274
Belgium	194	-0.0161
Pakistan	184	0.1474
Switzerland	165	0.146

## Discussion

To date, there have been no studies based on sentiment analysis on the use of intravenous or subcutaneous immunoglobulin therapy. Several studies on sentiment analysis relating to other treatments, such as the COVID vaccines and other therapies have been performed, but in spite of the large amounts of data on social media, the area of immunology is yet to catch up on using this tool (13, 15–17).

This study aimed to perform a sentiment analysis on tweets related to intravenous and subcutaneous immunoglobulin therapy from January 1, 2012, to December 31, 2022. Previous studies have focused on assessing the patient satisfaction and quality of life with both therapies and comparing them, but no consensus on which is best has been completely agreed upon due to both of them having their own caveats (18). Our study found a mean polarity for tweets related to intravenous immunoglobulin of 0.1295, while subcutaneous immunoglobulin related tweets had a mean polarity of 0.2117. Although both therapies present an overall positive sentiment, there is a much more positive sentiment on tweets relating to subcutaneous immunoglobulin. In a previous study, intravenous immunoglobulin therapy had mild side effects (headache, malaise, myalgia, fatigue, arthralgia, and low grade fever) in 5-25% of patients, while severe side effects were present in 1-5% of patients, with anaphylactic reactions in less than 1% of patients. In contrast, side effects for subcutaneous immunoglobulin therapy were milder and less frequent, but patients had more local adverse reactions (19).

In 2021, a study performed on 29 children with primary immunodeficiencies comparing quality of life and efficacy between intravenous and subcutaneous immunoglobulin therapy was performed, finding no significant difference in efficacy, but a reduction in missed daily activities in patients under subcutaneous therapy, which highly increased their quality of life (20). Incidence of adverse effects due to immunoglobulin varies widely throughout studies, this mostly due to study design variations, immunoglobulin preparations, and individual differences. Even though a certain conclusion on the incidence in side effects cannot be properly made, most studies point towards subcutaneous immunoglobulin therapy having less adverse effects (3, 21, 22).

In a 2020 study comparing intravenous and subcutaneous immunoglobulin therapy, a hypothetical cost analysis was conducted. The analysis demonstrated that the subcutaneous regimen yielded a total savings of 900 dollars compared to intravenous immunoglobulin. This finding provides an additional potential reason for patient preference toward subcutaneous immunoglobulin, further supporting the generally more positive sentiment identified in our analysis (23). In view of the evidence previously delve into, the lower amount of adverse effects, higher quality of life, and lower cost of subcutaneous immunoglobulin therapy could explain the overall more positive sentiment towards the subcutaneous immunoglobulin.

Regarding patient satisfaction, a 2015 study compared the use of subcutaneous immunoglobulin therapy to intravenous immunoglobulin therapy. Only seven patients responded to the survey, which was scored from 0 to 100, with 0 representing a preference for intravenous therapy and 100 representing a preference for subcutaneous therapy. The overall preference was strongly in favor of subcutaneous immunoglobulin, with a mean score of 93 (24). In a 2018 systematic review on the use of subcutaneous and intravenous immunoglobulin treatment in neuromuscular diseases, the 36-Item Short Form Survey (SF-36) scores from 49 patients and the Life Quality Index (LQI) scores from 115 patients were collected. The SF-36 scores showed a significant improvement in quality of life, with a mean score difference of 1.602 (95% CI [0.711–2.494], *p* < 0.0001). The LQI demonstrated a significant preference for subcutaneous therapy, with a mean difference of 17.80 (95% CI [16.152–19.420], *p* < 0.0001) (25).

Although the preference for subcutaneous immunoglobulin therapy has been demonstrated in previous studies using surveys, the analysis of social media data through natural language processing techniques offers a broader set of opinions in a more open environment. Therefore, this study provides a new and wider perspective on patients’ perceptions of the two types of treatment. Regarding the number of tweets, there was an overall trend towards more tweets in recent years, this could point toward an indication of more discussion in the general public towards immunoglobulin therapy. Nonetheless, the explanation behind this increase throughout the years is beyond the scope of this study.

Limitations of our study include, but are not limited to, a smaller sample size of tweets related to subcutaneous immunoglobulin therapy, the inability of the query search to distinguish between facilitated and conventional subcutaneous immunoglobulin therapy, the restriction of data to a single social media platform, the inability to determine if data originated directly from patients, and the use of only the English language, which limits input from countries where English is not the primary language. For future studies, the inclusion of other languages, as well as other social media sites would widen the global perspective relating to the use of these therapies and augment the amount of texts relating to subcutaneous immunoglobulin treatment.

## Conclusions

Social media provides a valuable platform for patients to discuss their treatments in a free environment, these discussions offer valuable data to better understand the patients perspectives. The present study performed a sentiment analysis on English-language tweets regarding immunoglobulin therapy over an 11 year period, analyzed from a temporal and geographical perspective. Results demonstrate an overall positive mean polarity in the 43,700 tweets regarding both subcutaneous and intravenous texts. Tweets linked to subcutaneous immunoglobulin treatment had a higher, and therefore, more positive mean polarity (0.2117) throughout the years, indicating a more positive public view and possibly a preference over intravenous immunoglobulin treatment. The amount of tweets relating to both therapies showed a tendency to increase as the years progressed, implying an increase in public discussion related to immunoglobulin treatment.

## Data Availability

The datasets presented in this article are not readily available because Data was obtained in accordance with Twitter´s policies at that moment. Requests to access the datasets should be directed to alextarango2001@gmail.com.

## References

[1] Yazdani R Habibi S Sharifi L Azizi G Abolhassani H Olbrich P. Common variable immunodeficiency: epidemiology, pathogenesis, clinical manifestations, diagnosis, classification, and management. J Investig Allergol Clin Immunol. (2020) 30:14–34. doi: 10.18176/jiaci.0388 30741636

[2] Amaya-Uribe L Rojas M Azizi G Anaya J Gershwin ME. Primary immunodeficiency and autoimmunity: A comprehensive review. J Autoimmun. (2019) 99:52–72. doi: 10.1016/j.jaut.2019.01.011 30795880

[3] Ness S. Differentiating characteristics and evaluating intravenous and subcutaneous immunoglobulin. Am J Manag Care. (2019) 25:S98–S104. 31318515

[4] Ballow M. Practical aspects of immunoglobulin replacement. Ann Allergy Asthma Immunol. (2017) 119:299–303. doi: 10.1016/j.anai.2017.07.020 28958372

[5] Borte M Hanitsch LG Mahlaoui N Fasshauer M Huscher D Speletas M. Facilitated subcutaneous immunoglobulin treatment in patients with immunodeficiencies: the FIGARO study. J Clin Immunol. (2023) 43:1259–71. doi: 10.1007/s10875-023-01470-2 PMC10088636 37036560

[6] Nandwani P Verma R. A review on sentiment analysis and emotion detection from text. Soc Netw Anal Min. (2021) 11:81. doi: 10.1007/s13278-021-00776-6 34484462 PMC8402961

[7] Barbounaki SG Gourounti G Sarantaki A. Advances of sentiment analysis applications in obstetrics/gynecology and midwifery. Mater Sociomed. (2021) 33:225–30. doi: 10.5455/msm.2021.33.225-230 PMC8563056 34759782

[8] Gong X Ying W Zhong S Gong S. Text sentiment analysis based on transformer and augmentation. Front Psychol. (2022) 13:906061. doi: 10.3389/fpsyg.2022.906061 35645894 PMC9136405

[9] Babu NV Kanaga GM. Sentiment analysis in social media data for depression detection using artificial intelligence: A review. SN Comput Sci. (2022) 3:74. doi: 10.1007/s42979-021-00958-1 34816124 PMC8603338

[10] Sarirete A. Sentiment analysis tracking of COVID-19 vaccine through tweets. J Ambient Intell Humaniz Comput. (2022) 30:1–9. doi: 10.1007/s12652-022-03805-0 PMC8966855 35378971

[11] Marques T Cezário S Lacerda J Pinto R Silva L Santana O. Sentiment analysis in understanding the potential of online news in the public health crisis response. Int J Environ Res Public Health. (2022) 19:16801. doi: 10.3390/ijerph192416801 36554680 PMC9779517

[12] van Draanen J Tao H Gupta S Liu S. Geographic differences in cannabis conversations on twitter: infodemiology study. JMIR Public Health Surveill. (2020) 6:e18540. doi: 10.2196/18540 33016888 PMC7573699

[13] Liu S Liu J. Public attitudes toward COVID-19 vaccines on English-language Twitter: A sentiment analysis. Vaccine. (2021) 39:5499–505. doi: 10.1016/j.vaccine.2021.08.058 PMC8439574 34452774

[14] R Core Team. R: A language and environment for statistical computing. Vienna, Austria: R Foundation for Statistical Computing (2022). Available at: https://www.R-project.org/.

[15] Austin MA Saxena A O'Malley TJ Maynes EJ Moncure H Ott N. Computational sentiment analysis of an online left ventricular assist device support forum: positivity predominates. Ann Cardiothorac Surg. (2021) 10:375–82. doi: 10.21037/acs-2020-cfmcs-fs-11 PMC8185377 34159118

[16] Lu TJ Nguyen AX Trinh X Wu AY. Sentiment analysis surrounding blepharoplasty in online health forums. Plast Reconstr Surg Glob Open. (2022) 10:e4213. doi: 10.1097/GOX.0000000000004213 35492229 PMC9038503

[17] Sharma C Whittle S Haghighi PD Burstein F Keen H. Sentiment analysis of social media posts on pharmacotherapy: A scoping review. Pharmacol Res Perspect. (2020) 8:e00640. doi: 10.1002/prp2.640 32813329 PMC7437347

[18] Achenbach CV Hernandez GH Gunten SV. The choice between intravenous and subcutaneous immunoglobulins: aspects for consideration. Pharmacology. (2022) 107:556–63. doi: 10.1159/000527655 36349790

[19] Bonilla F. Intravenous and subcutaneous immunoglobulin G replacement therapy. Allergy Asthma Proc. (2016) 37:426–31. doi: 10.2500/aap.2016.37.3987 27931296

[20] Sarı G Bilgin BG Yılmaz E Aytac G Karaca NE Aksu G. Efficacy and quality of life assessment in the use of subcutaneous immunoglobulin treatment for children with primary immunodeficiency disorder. Eur Ann Allergy Clin Immunol. (2021) 53:177–84. doi: 10.23822/EurAnnACI.1764-1489.179 33191716

[21] Guo Y Tian X Wang X Xiao Z. Adverse effects of immunoglobulin therapy. Front Immunol. (2018) 9:1299. doi: 10.3389/fimmu.2018.01299 29951056 PMC6008653

[22] Shabaninejad H Asgharzadeh A Rezaei N Rezapoor A. A comparative study of intravenous immunoglobulin and subcutaneous immunoglobulin in adult patients with primary immunodeficiency diseases: A systematic review and meta-analysis. Expert Rev Clin Immunol. (2016) 12:595–602. doi: 10.1586/1744666X.2016.1155452 26902306

[23] Allen JA Gelinas DF Freimer M Runken MC Wolfe GI. Immunoglobulin administration for the treatment of CIDP: IVIG or SCIG? J Neurol Sci. (2020) 408:116497. doi: 10.1016/j.jns.2019.116497 31765922

[24] Hadden RD Marreno F. Switch from intravenous to subcutaneous immunoglobulin in CIDP and MMN: improved tolerability and patient satisfaction. Ther Adv Neurol Disord. (2015) 8:14–9. doi: 10.1177/1756285614563056 PMC4286942 25584070

[25] Sala TP Crave J Duracinsky M Bompeka FL Tadmouri A Chassany O. Efficacy and patient satisfaction in the use of subcutaneous immunoglobulin immunotherapy for the treatment of auto-immune neuromuscular diseases. Autoimmun Rev. (2018) 17:873–81. doi: 10.1016/j.autrev.2018.03.010 30005853

